# Editorial: Targeting metabolism to activate T cells and enhance the efficacy of checkpoint blockade immunotherapy in solid tumors

**DOI:** 10.3389/fimmu.2023.1247178

**Published:** 2023-07-27

**Authors:** Zhijia Xia, Shi Chen, Miao He, Benhua Li, Yayuan Deng, Lin Yi, Xiaosong Li

**Affiliations:** ^1^ Department of General, Visceral, and Transplant Surgery, Ludwig-Maximilians-University Munich, Munich, Germany; ^2^ Clinical Molecular Medicine Testing Center, The First Affiliated Hospital of Chongqing Medical University, Chongqing, China; ^3^ Laboratory Animal Center, Chongqing Medical University, Chongqing, China; ^4^ Key Laboratory of Major Brain Disease and Aging Research (Ministry of Education), Institute for Brain Science and Disease, Chongqing Medical University, Chongqing, China

**Keywords:** metabolism, T cells, checkpoint blockade, immunotherapy, solid tumors

## 1 Introduction

In recent decades, immunotherapy has emerged as a groundbreaking approach in cancer treatment, offering new hope for patients. However, its efficacy remains limited due to low response rates and significant variations among individuals. One promising avenue to improve the effectiveness of immunotherapy is targeting the metabolism of cancer cells and the immune microenvironment. Recent studies have demonstrated the potential of metabolic interventions in controlling tumor progression and enhancing the immune response. T cells, an integral component of the immune system, play a critical role in the control and elimination of tumor cells. Recent studies have highlighted the influence of metabolic regulation on T cell activation and function.

This editorial aims to shed light on this topic by discussing some of the important articles published on the subject. These articles delve into various aspects, including the role of tumor-associated fibroblasts (CAFs), T cell exhaustion, the connection between inflammatory factors and T cell metabolism, as well as the impact on immunotherapy.

### 1.1 Tumor-associated fibroblasts (CAFs)

Under normal metabolic conditions, T cells primarily rely on oxidative phosphorylation for energy production. However, in the tumor microenvironment, highly active CAFs release a significant amount of lactate, leading to acidification of the tumor tissue (1). The acidic tumor microenvironment inhibits the activation and function of T cells, reducing the effectiveness of immune checkpoint blockade therapies. Therefore, inhibiting CAFs can activate T cells by modulating their metabolism, particularly by reducing lactate production and enhancing T cell oxidative phosphorylation metabolism. This activation of T cells can enhance their immune response against tumors. This suggests that different CAF activities may result in variations in clinical outcomes for cancer patients and differences in responses to immunotherapy. In a study by Xie et al., a CAF-related Prognostic Index (CPI) was established based on CAF-related genes. The study found that TNBC (triple-negative breast cancer) patients with a high CPI exhibited significantly reduced infiltration of CD8+ T cells and increased infiltration of Tregs within the tumor tissue. This CPI index accurately predicted the prognosis of TNBC patients and demonstrated correlation with immunotherapy responsiveness. These findings emphasize the significance of understanding CAFs in cancer and provide potential strategies for improving the effectiveness of immunotherapy (2). However, further research is needed to explore the association between the CPI index and metabolism, such as lactate metabolism levels. This understanding would enable the assessment of the metabolic status of cancer tissues based on the CPI index and facilitate the development of more precise treatment strategies. For instance, by targeting CAFs to reduce lactate levels, enhance oxidative phosphorylation metabolism, and improve the acid-base balance in tumor tissues, T cell activation and function can be augmented, ultimately enhancing the response to immunotherapy.

### 1.2 T cell exhaustion

T cell exhaustion represents a significant hallmark of immune responses and is frequently observed in chronic infections and immune-related disorders, including cancer. T cells serve as the principal effectors of the immune system, responsible for the identification and elimination of aberrant cells. However, under prolonged exposure to chronic stimuli or within the tumor microenvironment, the functionality and vitality of T cells gradually diminish, accompanied by a loss of memory T cell characteristics, leading to the development of exhaustion phenomena. The distinguishing features of T cell exhaustion encompass the overexpression of inhibitory surface receptors, such as programmed cell death protein 1 (PD-1) and cytotoxic T lymphocyte-associated antigen 4 (CTLA-4), along with aberrant cellular functions, including compromised cell proliferation and reduced capacity to generate cytotoxic molecules. These aberrations culminate in immune suppression, impeding the ability of T cells to effectively target cancer cells and curtailing the efficacy of immunotherapy. Notably, under exhausted conditions, T cells tend to transition from oxidative phosphorylation as a metabolic pathway to alternative routes like glycolysis and fatty acid oxidation. These metabolic adaptations result in inadequate energy supply, consequently impairing T cell functionality. Recent investigations have demonstrated that by modulating T cell metabolism, particularly by targeting glycolysis and oxidative phosphorylation pathways, it is possible to effectively reverse T cell exhaustion (3). This intervention not only enhances the anti-tumor potential of T cells but also improves the therapeutic outcome of checkpoint blockade immunotherapy in the treatment of solid tumors (4). These findings suggest that the extent of T cell exhaustion may exert an influence on the response of cancer patients to immunotherapeutic interventions. In a study by Chi et al., the phenomenon of T-cell depletion (TEX) and its impact on the response to immune therapy in hepatocellular carcinoma (HCC) were explored. The authors observed that HCC patients exhibiting a high TEX index displayed upregulation of nearly all immune checkpoints, except for CD40LG, thus contributing to variations in response to immunotherapy among patients. Consequently, targeting metabolic therapies may offer a promising approach to enhance the immune responsiveness of HCC patients with elevated TEX levels.

### 1.3 Inflammatory factors

The dysregulation of inflammatory factors contributes to aberrant T cell metabolism. Through various pathways, inflammatory factors exert regulatory control over T cell metabolic processes, thereby exerting an impact on immune responses. Firstly, certain inflammatory factors upregulate the expression of glucose transporter 1 (Glut1), glycolytic enzymes, and critical regulatory factors, thereby facilitating glucose uptake and promoting lactate production in CD8+ T cells, consequently driving the activation of effector T cells (5, 6). Secondly, inflammatory factors also influence the fatty acid oxidation pathways of T cells, thereby governing T cell memory (7). Furthermore, inflammatory factors can modulate amino acid metabolism and the tricarboxylic acid cycle of T cells, ultimately influencing their proliferation and functionality. In a study by He et al., the predictive capacity of interleukin-1 (IL-1) for immune response in lung adenocarcinoma (LUAD) patients was investigated. The researchers observed that LUAD patients with elevated levels of IL-1 exhibited a superior immune response. Single-cell transcriptomic analysis unveiled that IL-1 signaling contributes to the promotion of antigen presentation and correlates with the infiltration of activated CD8 T cells. This effect may be associated with the induction of glycolytic activity in T cells following IL-1 treatment (5). Additionally, Alshaebi et al. provided a comprehensive overview of the impact of IL-34, a human protein discovered in 2008, on immune checkpoint inhibitor therapy. However, although existing studies have demonstrated the ability of IL-34 to regulate immune cell function through metabolic reprogramming (8), research on the influence of IL-34 on T cell metabolism remains limited. Future investigations in this field are warranted to elucidate the effects of IL-34 on T cell metabolism, thereby facilitating the development of novel therapeutic strategies for immunotherapy.

### 1.4 Cancer cell metabolism

Metabolic reprogramming in tumor cells is a critical characteristic of tumor initiation and progression (9). One extensively studied metabolic abnormality in tumor cells is aerobic glycolysis, also known as the Warburg effect. Additionally, tumor cells exhibit aberrant lipid and amino acid metabolism. These metabolic alterations provide survival and proliferative advantages to tumor cells while also influencing the tumor microenvironment (TME). These metabolic changes collectively disrupt the activity of T cells. Ganjoo et al. provide a comprehensive overview of the intricate interplay between tumor metabolism and T-cell dysfunction: (1) the abnormally high glycolytic levels in tumor cells result in TME acidification, lactate accumulation, hypoxia, and glucose deprivation. These conditions upregulate PD-L1 expression in cancer cells and promote CTL apoptosis, significantly impairing T-cell activity; (2) under glucose-depleted conditions in the TME, CD8+ tumor-infiltrating lymphocytes (TILs) often rely on fatty acids as alternative energy sources to enhance their anti-tumor activity. However, tumor cells acquire lipids from the extracellular environment, depleting the energy sources available to T cells and compromising their activity; (3) tumor cells compete with T cells for essential and nonessential amino acids in the extracellular environment, resulting in suppressed T-cell activity. In summary, tumor cells exploit various metabolic pathways that lead to the consumption or accumulation of key metabolites, significantly impairing T-cell activity and suppressing immune responses. Targeting critical metabolic enzymes is crucial for restoring T-cell activity. Sphingolipids, a category of lipids, play a significant role in tumor cell proliferation (10). Zhang et al. stratified LUAD patients based on sphingolipid levels and observed a significant enrichment of the glycolysis pathway in the high-scoring group, along with lower expression of immune checkpoint markers. This suggests that immune vaccination, in combination with strategies targeting the glycolysis pathway, may be more suitable for LUAD patients with high sphingolipid scores, as opposed to immune checkpoint blockade (ICB) alone.

### 1.5 T-cell metabolism

As previously mentioned, nutrient metabolism is not only crucial for sustaining proliferation in tumor cells but also essential for maintaining robust immune activity in T cells. Chen et al. extensively describe the impact of T-cell metabolism on immunotherapy. A notable phenomenon is that rapidly proliferating T cells exhibit metabolic reprogramming characteristics similar to cancer cells. This partially explains why metabolic hyperactivity in tumor cells leads to diminished T-cell activity. Furthermore, Chen et al. summarize the persistent metabolic impairments observed in T cells across various anti-tumor immunotherapies, significantly impacting the anti-tumor function of cytotoxic T cells. Consequently, targeting T-cell metabolism in combination with immunotherapy represents a promising anti-tumor strategy.

## 2 Conclusion and perspectives

Collectively, these studies highlight the significance of metabolic interventions in tumor immunotherapy and provide insights into the development of personalized immunotherapies. By comprehending the metabolic profiles of individual patients and mapping out the cancer immunity atlas, these studies pave the way for specific immune therapies or combinations of immune therapies tailored to each patient’s unique characteristics. It is crucial to emphasize that clinical validation and biological verification of these findings are imperative for their successful translation into effective clinical strategies. Therefore, further validation studies should be included in the future.

In conclusion, the integration of metabolism and immunotherapy represents a promising approach to combat solid tumors. By targeting cancer metabolism and reshaping the immunosuppressive microenvironment, we can better harness the power of the immune system to attack cancer cells.

## Author contributions

ZX, SC, MH and BL drafted the manuscript. YD and LY revised the manuscript. XL conceived and helped with the final revision of this manuscript. All authors reviewed and approved the final manuscript.
